# Optimal Strategies for Colorectal Cancer Screening

**DOI:** 10.1007/s11864-022-00962-4

**Published:** 2022-03-22

**Authors:** Shailavi Jain, Jetrina Maque, Artin Galoosian, Antonia Osuna-Garcia, Folasade P. May

**Affiliations:** 1 Department of Medicine, David Geffen School of Medicine, UCLA Ronald Reagan Medical Center, University of California Los Angeles, 757 Westwood Plaza, Los Angeles, CA 90095 USA; 2 Vatche and Tamar Manoukian Division of Digestive Diseases, Department of Medicine, David Geffen School of Medicine, University of California Los Angeles, 650 S. Charles E Young Drive, Center for Health Sciences, Suite A2-125, Los Angeles, CA 90095-6900 USA; 3 Louise M. Darling Biomedical Library, University of California, Los Angeles, Center for Health Sciences, 12-077, Los Angeles, CA 90095-1798 USA; 4 Greater Los Angeles Veterans Affairs Healthcare System, Los Angeles, CA USA; 5 UCLA Kaiser Permanente Center for Health Equity, Jonsson Comprehensive Cancer Center, 650 S. Charles E Young Drive, Center for Health Sciences, Suite A2-125, Los Angeles, CA 90095-6900 USA

**Keywords:** colorectal cancer screening, fecal immunochemical test, sigmoidoscopy, colonoscopy, multi-target stool DNA, CT colonography, liquid biopsy

## Abstract

Colorectal cancer (CRC) imposes significant morbidity and mortality, yet it is also largely preventable with evidence-based screening strategies. In May 2021, the US Preventive Services Task Force updated guidance, recommending screening begin at age 45 for average-risk individuals to reduce CRC incidence and mortality in the United States (US). The Task Force recommends screening with one of several screening strategies: high-sensitivity guaiac fecal occult blood test (HSgFOBT), fecal immunochemical test (FIT), multi-target stool DNA (mt-sDNA) test, computed tomographic (CT) colonography (virtual colonoscopy), flexible sigmoidoscopy, flexible sigmoidoscopy with FIT, or traditional colonoscopy. In addition to these recommended options, there are several emerging and novel CRC screening modalities that are not yet approved for first-line screening in average-risk individuals. These include blood-based screening or “liquid biopsy,” colon capsule endoscopy, urinary metabolomics, and stool-based microbiome testing for the detection of colorectal polyps and/or CRC. In order to maximize CRC screening uptake in the US, patients and providers should engage in informed decision-making about the benefits and limitations of recommended screening options to determine the most appropriate screening test. Factors to consider include the invasiveness of the test, test performance, screening interval, accessibility, and cost. In addition, health systems should have a programmatic approach to CRC screening, which may include evidence-based strategies such as patient education, provider education, mailed screening outreach, and/or patient navigation, to maximize screening participation.

## Introduction

Colorectal cancer (CRC) is the third most common cancer and the second leading cause of cancer-related deaths in the United States (US) [1••] . While there has been an overall decline in the number of CRC cases since the 1980s, incidence has been increasing in individuals under the age of 50 since the early 1990s [2]. Most CRCs develop through the adenoma-carcinoma sequence and can be identified with screening tests that aim to detect precancerous polyps (e.g., advanced adenomas), early cancers, or both [3••].

The US Preventive Services Task Force (USPSTF) recommends screening for all average-risk adults from age 45 to 49 (grade B recommendation) and from age 50 to 75 (grade A recommendation) by one of many screening strategies that vary on scientific approach, invasiveness, frequency of testing, cost, and availability [1, 4]. Modeling studies have shown that each of these screening modalities has the potential to increase life expectancy [5•]. Nonetheless, only 67.1% of adults age 50–75 were up-to-date with CRC screening in the US in 2019 [6], and screening rates vary by age, geographic region, insurance status, race/ethnicity, socioeconomic status, educational achievement, and source of healthcare [7]. There are also newer CRC screening modalities that are not yet recommended first line for average-risk individuals. In this piece, we summarize the test characteristics, advantages, disadvantages, and clinical practice considerations for currently available and emerging CRC screening modalities.

## Screening modalities

The USPSTF currently recommends seven strategies for CRC screening. The stool-based CRC screening tests include the high-sensitivity guaiac fecal occult blood test (HSgFOBT), fecal immunochemical test (FIT), and multi-target stool DNA (mt-sDNA) test. The direct visualization screening tests include computed tomographic (CT) colonography, flexible sigmoidoscopy, and traditional colonoscopy. The seventh strategy is flexible sigmoidoscopy with a FIT, which combines a stool-based test and direct visualization [4••]. Emerging screening tests include colon capsule endoscopy (CCE), blood-based screening tests, stool-based microbiome studies, and urinary metabolomics.

### Stool-based screening modalities

#### High-sensitivity guaiac-based fecal occult blood test

The HSgFOBT is a stool-based screening modality that aims to detect colorectal polyps and cancers through an oxidation reaction. When heme is present in a stool sample, the alpha-guaiaconic acid contained on the testing card is oxidized by the hydrogen peroxide reagent to create a blue color, representing an abnormal (positive) result [4, 8]. This screening strategy requires individuals to collect and submit three consecutive stool samples annually, and any one abnormal result warrants a colonoscopy to evaluate for colorectal polyps or cancers.

There is limited new data on HSgFOBT in the US in recent years due to heavy reliance on colonoscopy and other stool-based screening tests. The 2021 systematic review commissioned by the USPSTF reported a sensitivity for CRC of 0.50 to 0.75 (95% CI, 0.09–1.0) and specificity of 0.96–0.98 (95% CI, 0.95–0.99) (Hemoccult Sensa, Beckman Coulter) (Table 1) [1••]. Sensitivity for advanced adenomas is lower at 0.06–0.17 (95% CI, 0.02–0.23), while specificity for advanced adenomas is 0.96–0.99 (95% CI, 0.96–0.99) (Hemoccult Sensa, Beckman Coulter) [1••]. A 2019 meta-analysis of six trials over a 15-year period demonstrated that screening with gFOBT led to a reduction in CRC-related mortality (annual: RR 0.69; 95% CI, 0.56–0.86) but did not reduce CRC incidence (annual: RR 0.86; 95% CI, 0.72–1.03) [9•].

**Table 1:** Summary of the efficacy, cost effectiveness, and patient adherence of CRC screening modalities

Screening method	Specificity	Sensitivity	Adherence	Lifetime number of tests needed per 1000 individuals screened**	Cost-effectiveness
**Stool-based strategies recommended by the USPSTF**					
High-sensitivity guaiac fecal occult blood test (HSgFOBT)	96–98% for CRC [1••]	50–75% for CRC [1••]	40–67% [55, 62]	Annual testing: 21,612 [4••]	Lower cost compared to colonoscopy [63]
Fecal immunochemical test (FIT)	94% for CRC [1••]	74% for CRC [1••]	31–73% [47, 64, 65]	Annual testing: 21,094 [4••]	Lower cost compared to CT colonography, colonoscopy, capsule endoscopy and mt-sDNA [11, 66]
Multi-target stool DNA (mt-sDNA) test	85% for CRC [1••]	93% for CRC [1••]	~75% [17]	Annual testing: 16,224 Q3y: 8,855 [5•]	Higher cost compared to FIT [22, 66]
**Direct visualization techniques recommended by the USPSTF**					
Computed tomography (CT) colonography	94% for adenomas ≥10 mm [1••]	89% for adenomas ≥10 mm [1••]	30–34% [67, 68]	Q5y: 6,609 [4••, 5•]	Lower cost compared to colonoscopy [69]
Flexible sigmoidoscopy	83–94% for proximal colon advanced neoplasms [70]	90–100% for distal colon CRC [3••]	27% [67]	Q5y: 6,563 [5•]	Lower cost compared to colonoscopy [30]
Colonoscopy	89% for adenomas ≥10 mm [1••]	18–100% for CRC [1••]	22–38% [55, 67, 68, 71]	Q10y: 4,248 [5•]	Higher cost compared to stool screening and other direct visualization tests [11]
**Emerging technologies (not currently USPSTF recommended)**					
Colon capsule endoscopy	91% for advanced neoplasia ≥10 mm [4••]	77% for advanced neoplasia ≥10 mm [4••]	80–90% after positive FIT [63]	Q5y: 2,736 colonoscopies/1,000 people, Q10y: 2,173 colonoscopies/1,000 people [66]	Approximately twice the cost of colonoscopy [40]
Liquid biopsy—methylated DNA (Epi ProColon)	79% for CRC [41]	68% for CRC [41]	83% [71]	Not known	Projected to have similar costs to mt-sDNA [72]
Liquid biopsy—methylated DNA (TriMeth)	99% for CRC [42••]	80% for CRC [42••]	Not known	Not known	Not known
Liquid biopsy—miRNA	26% for CRC [43]	85% for CRC [43]	Not known	Not known	Not known
Stool-based microbiome tests	78% for CRC [48]	62-78% for CRC [48]	Not known	Not known	Not known
Urine-based screening tests	80-96% for CRC [49, 51]	80-100% for CRC [49, 51]	Not known	Not known	Not known

**Tests include high-sensitivity guaiac fecal occult blood test (HSgFOBT), fecal immunochemical test (FIT), multi-target stool DNA (mt-sDNA)-FIT, CT colonography, flexible sigmoidoscopy, and colonoscopy and accounts for additional colonoscopies required for positive results from other screening modalities. Estimate is based on CISNET modeling study assuming 100% adherence and screening starting at age 45 years. Estimates from Davidson, KW, et al. [4••]

“Not known”: No data available

Abbreviations: *USPSTF* United States Preventive Services Task Force, *CRC* colorectal cancer, *Q3y* every 3 years, *Q5y* every 5 years, *Q10y* every 10 years

The benefits of this screening modality are that it is inexpensive, widely available, non-invasive, and can be performed outside of clinical settings. However, it does require dietary and medication restrictions. Many foods (i.e., red meat, rare meat, raw beets, carrots, cauliflower, cucumbers, grapefruit, mushrooms, broccoli, radish, horseradish, turnips) and medications (non-steriodal anti-inflammatory drugs, vitamin C, iron, vitamin E, blood thinners) must be avoided for 2 days prior to testing as they may cause an abnormal result. In addition, the test should not be performed in the presence of upper or lower gastrointestinal bleeding [8]. Although there are many different formulations of the FOBT, the 2021 USPSTF guidelines specifically recommend only newer high sensitivity formations (HSgFOBT) [1••].

#### Fecal Immunochemical Test

FIT is a stool-based screening test that uses an antibody assay to detect the presence of the intact globin portion of human hemoglobin in the stool [10]. There are numerous FIT products on the commercial market; however, the OC-Sensor test (Eiken Chemical) is commonly used due to its relatively high sensitivity and specificity [1••]**.** Based on the manufacturer’s recommended cut-off of 20μgHb/g feces, sensitivity and specificity for CRC are 0.74 (95% CI, 0.64–0.83) and 0.94 (95% CI, 0.93–0.96), respectively (Table 1) [1••]. Sensitivity and specificity for advanced adenoma are improved compared to HSgFOBT; sensitivity for advanced adenoma is 0.23 (95% CI, 0.20–0.25) and specificity is 0.96 (95% CI, 0.95–0.97) [1••]. A 2015 cohort study (*n*=5,417,699) demonstrated a reduction in CRC mortality with biennial FIT (RR 0.90, 95% CI, 0.84–0.95), but no change in CRC incidence [1••].

Unlike HSgFOBT, FIT requires only one stool sample annually and is not impacted by an individual’s diet or medications. In addition, it does not present an abnormal result in the presence of upper gastrointestinal bleeding as hemoglobin from foregut lesions is partially digested before it reaches the colon [10]. For these reasons and ease of use, it is currently the most common non-invasive CRC screening modality among average-risk individuals [7]. FIT is also highly accessible and less costly than screening colonoscopy and mt-sDNA [11]. In a 2020 comparative analysis, CRC detection rates were similar when four rounds of FIT every other year were compared to one-time flexible sigmoidoscopy and one-time colonoscopy [12•].

FIT does have its limitations, however. The 2021 USPSTF guidelines recommend that FIT be performed annually for CRC screening, which can require considerable healthcare resources for patient outreach [4••]. Like HSgFOBT and other non-colonoscopic screening modalities, it is a two-step screening strategy in which individuals with an abnormal result warrant a colonoscopy to complete the screening process [4••]. Colonoscopy completion rates after abnormal FIT are as low as 30% in some settings [13]. An additional focus is the appropriate cut-off value for an abnormal FIT in FIT-based population-based screening programs [3, 14–16]. Use of a low cut-off value (e.g.,10μgHgb/g) can increase sensitivity and positive predictive value but reduces specificity, requiring a larger number of follow-up colonoscopies and increasing the potential for adverse events during colonoscopy [14].

#### Multi-target stool DNA test

Cologuard (Exact Sciences) is a newer stool-based screening modality that aims to detect 11 molecular biomarkers for abnormal DNA (e.g., mutant *KRAS*, methylated *BMP3*, and methylated *NDRG4)*, including human hemoglobin via a FIT [3••]. An abnormal test result is determined by a calculated score based on the presence and quantity of each biomarker [17]. It is the only mt-sDNA test currently commercially available [1••]. Its sensitivity and specificity for CRC are 0.93 (95% CI, 0.87–1.0) and 0.85 (95% CI, 0.84–0.86), respectively (Table 1) [1••]. For advanced adenoma, sensitivity is 0.43 (95% CI, 0.40–0.46) and specificity is 0.89 (95% CI, 0.86–0.92) [1••]. A 2021 study demonstrated that with perfect adherence, mt-sDNA reduces CRC incidence by 66% (95% CI, 63–68%) [11].

The benefits of mt-sDNA are that it can be performed in non-clinical settings, does not require diet or medication restrictions, can be performed every 3 years, and is non-invasive with low potential for adverse effects [4, 18]. Additionally, there may be increased sensitivity for right-sided colon lesions that are more often missed with other non-colonoscopic screening methods [19]. Exact Sciences also provides electronic and live patient navigation to guide patients through the screening process and increase test completion rates [17].

Challenges with mt-sDNA screening include cost, a high false positive rate compared to FIT, and some uncertainty as to whether further diagnostic work-up is necessary when the result is positive but the follow-up colonoscopy is negative [11, 20]. Currently, in this situation, the recommendation is that asymptomatic individuals should not undergo additional testing or procedures [3••].

Overall, when comparing mt-sDNA screening to FIT, mt-sDNA is better at differentiating between advanced precancerous lesions (advanced adenomas and serrated polyps ≥10mm and/or with low- or high-grade dysplasia) and non-neoplastic or negative findings (*p*=0.004) [21]. It also has a higher sensitivity for advanced adenomas, large and serrated lesions, multiple lesions, large lesions, and tubulovillous lesions compared to FIT [3••]. However, specificity is lower than FIT, which can result in more colonoscopies, more adverse events, and higher costs [3, 4]. There is ongoing research to improve the sensitivity and specificity of the mt-sDNA test, and as test characteristics and insurance coverage improve, use of this screening strategy will likely increase [22].

### Direct visualization screening modalities

#### Computed tomographic colonography (virtual colonoscopy)

Virtual colonography uses a computed tomography (CT) scanner and computer reconstruction methods to visually evaluate the colon and rectum for colorectal polyps and cancers. For CRC screening, the recommended testing interval is 5 years, and individuals with abnormal findings must undergo traditional colonoscopy [23]. Sensitivity for adenomas 10mm or greater is 0.89 (95% CI, 0.83–0.96), and specificity is 0.94 (95% CI, 0.89–1.0) (Table 1) [1••]. For adenomas 6mm or larger, sensitivity is 0.86 (95% CI, 0.78–0.95), and specificity is 0.88 (95% CI, 0.83–0.95) [1••].

The benefits of CT colonography are that it is less invasive than colonoscopy, does not require sedation or anesthesia, has a low complication rate, and is relatively safe for individuals with medical comorbidities that preclude traditional colonoscopy [3, 24]. Compared to mt-sDNA, CT colonography detects more advanced adenomas and has a higher positive predictive value [18]. Unlike stool-based screening tests, it often allows for same-day endoscopic evaluation if needed [18]. Additionally, CT colonography can assess for invasive malignancy and the presence of metastasis [25] and has the potential to screen for other conditions, including poor bone mineral density, aortic calcification, and hepatic steatosis [18].

Potential unintended consequences of CT colonography include cumulative exposure of radiation with repeated examinations and the detection of incidental findings that require further work-up. The test results in clinically insignificant or indeterminate findings in 1.3–11.4% cases and potentially important findings in 3–4% of cases [1, 3, 4, 18]. Lack of colonoscopic follow-up after abnormal CT colonography is also a notable clinical challenge as with other forms of non-colonoscopic screening [23].

#### Flexible sigmoidoscopy

Flexible sigmoidoscopy is an endoscopic CRC screening modality that allows for direct visualization of the rectum, sigmoid colon, and descending colon. In addition, the procedure allows for resection or biopsy of distal colorectal lesions. Pooled data from four randomized control trials demonstrated a reduction in CRC incidence (IRR 0.78, 95% CI, 0.74–0.83) and CRC mortality (IRR 0.74, 95% CI, 0.68–0.80) with 1- or 2-time use of screening flexible sigmoidoscopy [26–29].

Benefits of flexible sigmoidoscopy over colonoscopy include reduced bleeding and perforation risk, avoidance of sedation and anesthesia, and lower cost [30, 31]. In addition, flexible sigmoidoscopy can be provided by a broader range of clinician specialists (e.g., primary care providers, family medicine providers).

The 2021 USPSTF guidelines recommend flexible sigmoidoscopy either alone every 5 years or every 10 years with an annual FIT [4••]. While flexible sigmoidoscopy every 5 years is associated with a similar number of life years gained as biennial FIT, the addition of FIT to flexible sigmoidoscopy results in a similar number of life years gained and similar mortality reduction to colonoscopy [5•]. The major limitations of flexible sigmoidoscopy are the inability to examine the entire colon (resulting in no incidence or mortality benefit for proximal CRCs) and low adherence [30]. Due to these limitations, flexible sigmoidoscopy (with or without FIT) is a less common strategy for CRC screening in the US and is largely reserved for clinical settings and populations with limited access to insurance, colonoscopy, and/or gastroenterologists.

#### Colonoscopy

Colonoscopy is the most common screening modality in the US and allows for visual examination of the entire colon and rectum for colorectal polyps and cancers. Sensitivity is 0.89–0.95 (95% CI, 0.70–0.99), and specificity is 0.89 (95% CI, 0.86–0.91) for adenomas 10mm or larger [1••]. For CRC, sensitivity is 0.18–1.0 (95% CI, 0.01–1.0) (Table 1) [1••].

In two large cohort studies, colonoscopy was associated with a reduction in CRC incidence (HR 0.53, 95% CI, 0.40–0.71) and a reduction in CRC-related mortality (HR 0.32, 95% CI, 0.24–0.45) [32, 33]. While studies consistently demonstrate a reduction in distal CRC incidence, the data are less consistent to support a reduction in incidence of proximal CRCs [30]. However, the overall reduction in CRC incidence and CRC-related mortality is greater for colonoscopy than for flexible sigmoidoscopy [30]. Additional benefits of colonoscopy are that it is a one-step definitive screening option, it is required only once every 10 years (if normal), and it allows for any detected lesions to be removed or biopsied.

Despite these benefits, colonoscopy requires bowel preparation and changes to diet and medication prior to the procedure. It is also seen by many patients as very invasive, causing patient hesitancy and non-adherence [1, 34, 35]. It is not available in some clinical settings, is more costly than the other recommended screening strategies, and is associated with a higher risk of complications [1, 11]. In a meta-analysis that included over 5 million colonoscopy procedures, serious adverse events included major bleeding (0.146%) and perforation (0.031%) [1••]. In addition, there is a high degree of inter-operator variability among gastroenterologists. An important and recent focus in CRC prevention and control has been to increase high-quality screening colonoscopy through the measurement, documentation, and improvement of several colonoscopy quality indicators that have been demonstrated to reduce development of interval CRCs [36–38].

### Novel emerging screening modalities

#### Colon capsule endoscopy

CCE is an emerging screening tool that involves ingestion of a pill-sized wireless camera that takes images as it travels through the gastrointestinal tract [3••]. Colonoscopy is required for definitive screening if images reveal colorectal polyps or cancers [3••]. Though not currently recommended by the USPSTF or other medical professional societies as first-line screening for average-risk individuals, CCE is approved by the US Food and Drug Administration (FDA) for CRC screening in individuals with a history of incomplete colonoscopic evaluation or with high risk of complications during colonoscopy [3, 39]. The more recent CCE-2 (second generation) has a sensitivity of 76.7% (95% CI, 63.7–86.2%) and specificity of 90.7% (95% CI, 83.6–95.0%) for advanced neoplasia 10mm or larger (PillCam, Medtronic) (Table 1) [39]. For this screening modality, sensitivity is dependent on the percentage of colon surface area imaged and the time to capsule excretion, with a transit time of 3–5 h showing a sensitivity of 100% for advanced neoplasia greater than 6mm [39].

CCE is minimally invasive; does not require sedation, insufflation, or radiation; and can be completed in a non-clinical setting [3••]. It performs better than CT colonography in detecting neoplasia greater than 6mm in individuals with a history of incomplete colonoscopy and has lower risk for serious adverse events than traditional colonoscopy [39].

However, the complete CCE examination rate is only 66.7% [40], and 32% of CCEs lead to colonoscopy referral (polyp cutoff of 10mm or larger) [39]. CCE interpretation also requires a clinician that is trained in reading capsule endoscopy and often takes more time than performing a traditional colonoscopy and preparing a report [40]. There is ongoing development of more accurate and less expensive CCE devices that can image a larger surface area of the colon and rectum. [39]. Improvements in test performance and lower cost may make CCE more feasible and acceptable as a first-line screening method in the future.

#### Blood-based screening tests (liquid biopsy)

Blood-based cancer detection tests, also known as “liquid biopsy,” represent a new area of potential for single-cancer or multiple-cancer detection strategies. CRC develops through an accumulation of both genetic and epigenetic alterations in the gut mucosa, which serve as candidates for the development of blood-based screening tests [3••]. Currently, there is only one FDA-approved blood-based CRC screening test (Epi proColon; Epigenomics AG, 2016), and none are recommended as first-line screening in average-risk adults. Epi proColon detects circulating methylated SEPT9DNA and had a sensitivity of 0.68 (90% CI, 0.53–0.80) and specificity of 0.79 (95% CI, 0.77–0.81) for CRC in one nested case-control study (*n*=6,857) (Table 1) [41]. Sensitivity and specificity for advanced adenomas are 0.22 (95% CI, 0.18–0.24) and 0.79 (95% CI, 0.76–0.82), respectively [41]. The TriMeth test is another DNA methylation test and aims to detect three CRC-specific DNA methylation markers (*C9orf50, KCNQ6*, and *CLIP4)*; it has a sensitivity of 0.80 and specificity of 0.99 for stage I CRCs in a recent discovery and validation study (CIs not provided) (Table 1) [42••].

Blood-based screening tests also include tests that detect plasma microRNA (miRNA) and plasma protein biomarkers. miRNA are expressed in the early phases of CRC development, are deregulated in precancerous and cancerous tissues, and remain relatively stable in the peripheral blood; therefore, they represent a unique area of interest in CRC screening [43]. In a 2020 analysis of 60 patients with an abnormal FIT result, the specificity of a combination of miRNAs was 0.19 for high grade adenomas and 0.26 for CRC when sensitivity was set at 0.85 (no CIs provided) (Table 1) [43]. Several plasma protein biomarkers (e.g., Interleukin-6, mannan-binding lectin serine protease 1, integrin alpha 11) are associated with CRC and are being actively evaluated in various combinations as potential screening strategies [44].

There is great interest in these tests given that they are minimally invasive and have the perceived potential for easy access and high patient adherence compared to the currently available screening methods [45]. However, factors yet to be determined include test accuracy, cost, and the appropriate follow-up after abnormal results. Additionally, the ability of these tests to detect precancerous polyps and CRC depends on the amount of tumor DNA shedding and the presence of circulating tumor cells and circulating tumor DNA in the peripheral blood. Unless high sensitivity is achieved with this technology, blood-based CRC screening can result in high numbers of false positive results, unnecessary colonoscopies, and adverse events during colonoscopy. In addition, the clinical implications of an abnormal blood-based screening test are uncertain when the follow-up colonoscopy is negative. It will be essential to determine the appropriate clinical work-up for other malignancies and conditions when colorectal polyps and CRC have been ruled-out. As with all innovation, blood-based screening tests must be evaluated in prospective, population-based studies to determine their accuracy at detecting colorectal polyps and cancers and their impact on CRC screening outcomes, including incidence, mortality, resource utilization, cost, and patient experience [46].

#### Stool-based microbiome tests

Stool-based microbiome tests are emerging screening modalities that are not yet FDA-approved or recommended for average-risk screening. The stool bacterial load can be higher in individuals with high-grade dysplasia and CRC, which has led to research to identify CRC bacterial markers in the stool [47]. However, given that these tests remain relatively new, there is limited data on test performance.

In a 2020 metagenomics and validation study, *Lachnoclostridium* sp. (*m3*), *Fusobacterium nucleatum* (*Fn*), and *Clostridium hathewayi* (*Ch*) were significantly enriched in adenoma [48]. At 78.5% specificity, fecal *m3* had 48.3% sensitivity for adenoma and 62.1% sensitivity for CRC (Table 1). Fecal *m3* performed better than *Fn* at identifying adenoma from controls (areas under the receiver operating characteristic curve (AUROCs) *m3*=0.675 vs *Fn*=0.620, *p*=0.09); however, *Fn* performed better at identifying CRC (AUROCs *Fn*=0.862 vs *m3*=0.741, *p*<0.0001). In a subgroup analysis in the same study, fecal *m3* had higher sensitivity than FIT for non-advanced and advanced adenomas (44.2% and 50.8% for *m3* vs. 0% and 16.1% for FIT). Overall, combining one or more fecal bacterial biomarkers with FIT optimized test performance; fecal *m3, Fn*, *Ch*, *Bacteroides clarus*, and FIT together had a sensitivity of 93.8% and specificity of 81.2% for CRC (no CIs provided) [48].

Like other stool-based screening modalities, stool-based microbiome tests will require follow-up colonoscopy when abnormal for definitive screening. An additional challenge with this screening strategy is that many current microbiome tests require genomic or metagenomic sequencing, which is time-consuming and expensive when compared to PCR tests [47]. Further research is needed to identify the optimal microbiome biomarkers and combinations of biomarkers and to evaluate test accuracy and cost.

#### Urine-based screening tests

Urine-based screening tests use liquid chromatography-mass spectrometry or nuclear magnetic resonance (NMR) spectroscopy to identify urine metabolites that are known to be associated with the presence of colorectal adenomas and CRC and represent another novel area in CRC screening [49, 50].

One study of a urine-based screening test that utilizes liquid chromatography-mass spectrometry found that diacetylspermine and kynurenine together in the urine had a specificity of 80.0% and sensitivity of 80.0% for CRC (no CIs provided) [49]. A study of a urine-based screening test that utilizes NMR spectroscopy identified a urine metabolomic profile with a sensitivity of 88.9% and specificity of 50.2% for adenomas [50]. Compared to gFOBT, this profile had a higher sensitivity for colorectal adenomas (size not specified) [50]. Finally, a recent study identified a urine metabolite profile that includes taurine, alanine, and 3-aminoisobutyrate and has a sensitivity of 75% and specificity of 100% for advanced adenomas and a sensitivity of 100% and specificity of 96.2% for CRC (no CIs provided) (Table 1) [51].

Some benefits of this screening modality are that it is non-invasive and samples can be collected in a non-clinical setting [49]. Additionally, NMR spectroscopy results are highly reproducible [51]. However, the current tests are limited by suboptimal sensitivity and specificity and their dependency on colonoscopy for definitive screening in the setting of an abnormal result [52]. Like most recently developed CRC screening modalities, there is no data on whether urine-based screening can impact CRC incidence and mortality, and they are not currently FDA-approved or formally recommended for CRC screening.

## Promoting optimal screening strategies

Despite strong evidence that CRC screening improves CRC-related outcomes, screening rates in the US continue to be far below the National Colorectal Cancer Roundtable goal of 80% screened in every community [53]. There is evidence that providing patients with a choice between various screening modalities can increase screening participation [54, 55], and the USPSTF and other organizations have emphasized that “the best test is the one that gets done.” As such, it is essential for patients and providers to engage in an informed shared decision-making process to discuss the advantages and disadvantages of the available screening strategies and to assess a patient’s individual CRC risk and screening preferences (Table 2). In these patient-provider interactions, patients’ lack of knowledge about CRC risk, misinformation about CRC screening tests, mistrust in the healthcare system, and barriers to screening can often be addressed to help encourage screening utilization.

**Table 2:** Advantages and disadvantages of recommended and emerging CRC screening modalities

Method	Advantages	Disadvantages
**Stool-based strategies recommended by the USPSTF**		
**High-sensitivity guaiac fecal occult blood test (HSgFOBT)**	• Mortality benefit in prospective longitudinal studies [1, 4, 9] • With perfect adherence, can achieve the most life-years gained compared to other screening tests [73] • Highly accessible and can be performed in non-clinical settings • Less invasive than direct visualization techniques • Low cost	• Abnormal test requires follow-up colonoscopy [3••] • Annual testing required [4••] • Dietary and medication restrictions prior to testing [4••] • Requires multiple stool samples each year [4••] • Should not be performed in presence of upper or lower gastrointestinal bleeding [8]
**Fecal immunochemical test (FIT)**	• Mortality benefit in retrospective studies [3••] • Increased participation compared to other modalities (colonoscopy, FOBT, sigmoidoscopy) [74] • Highly accessible and can be performed in non-clinical settings • Less invasive than direct visualization techniques • Low cost • No dietary restriction or bowel preparation required [3••] • Requires only one stool sample [3••] • Can be performed in setting of upper gastrointestinal bleeding [10] • Similar rates of CRC detection compared to flexible sigmoidoscopy	• Abnormal test requires follow-up colonoscopy [3••] • Annual testing required [3••] • Less sensitive for detecting CRC and adenomas than other modalities (CT colonography, capsule endoscopy, stool DNA) [66]
**Multi-target stool DNA (mt-sDNA) test**	• High participation (Exact Sciences patient navigation) [17] • Can be performed in non-clinical settings • Less invasive than direct visualization techniques • No dietary restriction or bowel preparation required [4, 18] • Requires only one stool sample [4••] • More sensitive that FIT alone [3••] • Testing can be performed every 3 years	• No data to support an incidence or mortality benefit [3••] • Abnormal test requires follow-up colonoscopy [3••] • Lower specificity compared to FIT resulting in more false positive results [3, 4] • Lower positive predictive value and detection rate for advanced adenomas compared to CT colonography [18] • High cost compared to other stool-based strategies [11]
**Direct visualization techniques recommended by the USPSTF**		
**Computed tomography (CT) colonography**	• Lower risk of complications compared to colonoscopy [3••] • Less invasive compared to colonoscopy [3••] • Lower cost compared to colonoscopy [69] • Does not require sedation [3••] • Can visualize the entire colon [4••] • Less frequent testing interval than stool-based modalities [23] • Relatively safe for individuals with medical comorbidities that preclude colonoscopy [3, 24] • Can allow for same day endoscopic evaluation if indicated [18] • High positive predictive value [18]	• No data to support an incidence or mortality benefit [4••] • Abnormal test requires follow-up colonoscopy [3••] • Requires dietary modification and bowel preparation [3••] • Less precise compared to other modalities [3••] • Requires exposure to radiation [3••]
**Flexible sigmoidoscopy**	• Mortality benefit when combined with annual FIT screening [4••] • Lower risk of complications compared to colonoscopy [4, 30] • Lower cost compared to colonoscopy [30] • Does not require sedation or oral bowel preparation [4, 30] • Allows for direct visualization of rectum, sigmoid colon and descending colon • Less frequent testing interval than FIT, HSgFOBT [4••] • Can be performed by broader range of clinicians than colonoscopy	• Studies show reduction in distal CRC incidence but no reduction in proximal CRC incidence [30] • Abnormal test requires follow-up colonoscopy [3••] • Requires per rectal bowel preparation (enema) [3••] • Does not examine entire colon [3, 4] • Low patient participation compared to stool- based screening strategies [30]
**Colonoscopy**	• Mortality benefit in retrospective studies [32, 33] • Allows for direct visualization of entire colon • Potential for least frequent testing interval • Allows for resection or biopsies of concerning lesions	• Invasive procedure typically performed under conscious sedation or anesthesia • Higher rate of complications compared to other direct visualization techniques [3••] • Requires bowel preparation and diet modification [4••] • Low accessibility in some populations and regions • Low patient participation [34] • Has a high degree of operator variability [3••] • Higher cost than other screening options [11]
**Emerging technologies (Not currently USPSTF recommended)**		
**Colon capsule endoscopy**	• Less invasive than direct visualization techniques [3••] • Does not require sedation [3••] • Can be performed in non-clinical settings [3••] • Lower risk of complications compared to colonoscopy [39]	• Not currently recommended by the USPSTF for CRC screening for average-risk individuals due to limited evidence [3••] • No data to support an incidence or mortality benefit • Abnormal result requires follow-up colonoscopy [3••] • Dietary restrictions and colon preparation may be required [4••] • Possibility of capsule retention in small bowel [3••] • Unclear ideal screening interval [3••] • Low accessibility in some populations and regions • Higher cost than colonoscopy [40] • Interpretation requires provider trained in reading capsule endoscopy [3••]
**Blood-based/liquid biopsy**	• Less invasive than direct visualization techniques [45] • No dietary restriction or bowel preparation required • Potential for broad availability and multiple cancer testing [39] • Will likely have high adherence compared to traditional methods [42••]	• Not currently recommended by the USPSTF for CRC screening for average-risk individuals due to limited evidence [3••] • No data to support an incidence or mortality benefit • Abnormal result requires follow-up colonoscopy [3••] • Only one test is currently FDA-approved (Epi proColon; Epigenomics AG, 2016) [41, 44] • Unclear cost and ideal testing interval
**Stool-based microbiome tests and urine-based tests**	• Less invasive than direct visualization techniques [49] • Can be performed in non-clinical settings [49] • Limited evidence showed greater sensitivity for adenomatous polyps compared to FIT (urine-based test) [50]	• Not currently recommended by the USPSTF for CRC screening for average-risk individuals due to limited evidence [3••] • No data to support an incidence or mortality benefit • Abnormal result requires follow-up colonoscopy [3••] • Low sensitivity and specificity compared to other techniques [48] • High cost due to genomic/metagenomic sequencing [47] • Unclear ideal testing interval • Does not distinguish by polyp size or stage of CRC [52]

Abbreviations: *USPSTF* United States Preventive Services Task Force, *CRC* colorectal cancer, *FDA* Food and Drug Administration

Beyond provider counseling, it is critical that health systems have organized strategies to encourage CRC screening. In the US, CRC screening is primarily opportunistic and only achieved if a primary care provider actively recommends it. Screening rates are higher in health systems that use evidence-based and programmatic approaches to screening, in which all patients who are eligible and due for screening receive automated interventions to maximize uptake. Some of the most effective evidence-based strategies include patient education [54•, 55•, 56•, 57•, 58•], provider education [54, 59], employing cadres of patient navigation [57, 60], and patient mailed outreach [54, 59–61]. Patient navigators help guide patients through the CRC screening process and the complexities of the health system, addressing educational, cultural, and logistical barriers that hinder screening completion and follow-up care while reducing burdens on primary care providers. Their use has been demonstrated to increase screening uptake by more than 100% [60]. Mailed outreach efforts include mailing FIT kits to patients due for screening, an approach that has also increased screening participation in population-based studies [13, 54, 57, 61]. Health systems with suboptimal CRC screening rates should be intentional in their population-health efforts and consider these population health strategies and others.

One of the main benefits of CRC screening is the broad range of available screening technologies, which offer great potential for both prevention and early detection. An understanding of the test characteristics, benefits, challenges, and clinical practice considerations for currently available and emerging CRC modalities is critical towards improving CRC screening uptake and CRC outcomes. In addition, further optimization of emerging screening technologies has the potential to decrease the overall burden of CRC in the US and globally.
